# Analysis of a SARS-CoV-2 daily screening programme for healthcare workers at a district hospital in KwaZulu-Natal, a quality improvement initiative

**DOI:** 10.4102/phcfm.v12i1.2525

**Published:** 2020-09-01

**Authors:** Amy Booth, Ridwaan A. Omed, Mergan Naidoo

**Affiliations:** 1 Department of Family Medicine, Faculty of Health Sciences, University of KwaZulu-Natal, Durban, South Africa

**Keywords:** SARS-CoV-2, COVID-19, healthcare workers, self-screening, occupational health, public health

## Abstract

The severe acute respiratory syndrome coronavirus 2 (SARS-CoV-2) has caused an unprecedented burden on our healthcare systems and workers. Healthcare workers are at risk of contracting and spreading SARS-CoV-2 given their proximity to positive cases, often with a lack of personal protective equipment. The South African Department of Health requires that all employees be screened daily for symptoms and potential persons under investigation identified timeously. This report aims to assesses the efficacy of daily self-screening tools in detecting and managing potential staff cases of SARS-CoV-2. Our hospital, situated in KwaZulu-Natal, South Africa, developed a daily self-screening tool for all healthcare workers to complete, consisting of questions on symptoms and epidemiological risk factors. The screening tools were collected and assessed after four weeks of use. Fifty-four forms were assessed. Twenty-eight (51.9%) forms were not completed, whilst 12 (22.2%) indicated positive symptoms with no documentation that any further medical assessment, testing or isolation was done. We identified that the poor completion of forms was likely because of the lack of education of staff on the importance of the forms, poor oversight by management, staff forgetfulness or lack of awareness of the forms. Screening of staff is vital during this pandemic but requires constant oversight by line managers, staff motivation and adequate education. Ongoing development of efficient screening programmes is required.

## Introduction

The severe acute respiratory syndrome coronavirus 2 (SARS-CoV-2), which causes coronavirus disease 2019 (COVID-19), has had a radical impact on healthcare systems and infection prevention control (IPC) globally. As of 07 May 2020, SARS-CoV-2 has infected 3 843 153 people and resulted in 265 657 deaths worldwide.^1^ Healthcare workers (HCWs) are at the forefront of this pandemic and a shortage of personal protective equipment (PPE), coupled with infrastructure shortfalls, limiting the capacity for patient isolation, places HCWs at significant risk of contracting SARS-CoV-2. As on 21 April 2020, 47 HCWs in the Western Cape tested positive, whilst 48 staff members from a private hospital in KwaZulu-Natal (KZN) contracted the virus.^2,3^ Given the risk that staff face, the South African Department of Health (DoH) issued guidelines for monitoring and managing employees.^4^ In a government gazette issued on 29 April 2020, it was mandatory for all employers to screen workers for any symptoms on commencement of duty.^5^ Our hospital in KZN had implemented many of these practices at the outset of the pandemic evolution. We adapted a daily screening tool for all staff members. The intention was to detect potential persons under investigation (PUIs), or SARS-CoV-2 suspects, among the staff and expedite further assessment at the occupational health clinic (OHC). This report outlines the screening programme and assesses its efficacy in detecting and managing potential staff cases of SARS-CoV-2.

## Method

All staff at our hospital were given the self-administered screening tool, which incorporated the PUI criteria (Appendix 1). The screening tool was adapted from the case definition of SARS-CoV-2 issued by the National Institute for Communicable Diseases at that time.^6^ Senior clinicians provided training for staff about the disease, demonstrated the correct use of PPE and familiarised them with the daily screening tool. If any staff member detected a positive response, they were to report to the on-site OHC for a full medical assessment, prior to reporting for duty. If the secondary assessment confirmed a positive screening outcome, there was a low threshold for testing PUIs and placing them in self-isolation. The recently released government gazette has since made it mandatory for any employee with symptoms to be excluded from entering the workplace and they must undergo immediate self-isolation and testing.^5^

The self-administered screening tools were first issued to staff on 30 March 2020, and an evaluation of its use was subsequently performed. The hospital consists of 15 wards, 6 outpatient departments and 3 outreach facilities. For the purpose of this report, only the screening tools of nursing staff were assessed as they were prioritised during training as high-risk frontline staff. A total of 273 nursing staff are employed at the hospital. Forms were sampled from 13 wards and 4 outpatient departments.

### Ethical consideration

This article followed all ethical standards for a research without direct contact with human or animal subjects.

## Results

Fifty-four forms were collected from the nursing staff on duty. Of these, 28 forms (51.9%) either had rows of the daily checklists incompletely filled or evidence of premature cessation. Twelve forms (22.2%) had evidence of positive symptoms, but there was no documentation indicating that these staff had been to the OHC or received any further assessment. Six wards reported that they were unaware of the tool, whilst one of the outpatient departments reported that staff declined completing the forms. Some staff reported that they forgot to complete the forms, were unaware that the forms would be collected or did not appreciate the importance of the forms. Only the day staff were there to hand in their forms when the collection was performed, largely excluding the night staff or those on leave. However, four of the wards and one of the outpatient departments kept all the forms together in a file and submitted both day and night staff tools.

## Discussion

The South African DoH has advised that all employees need to be screened for COVID-19 on a daily basis. We found that the method of screening by using a self-administered screening tool was ineffective. The poor completion of the screening tools could be a result of inadequate awareness and training around the tool or staff’s perceptions that they could screen themselves and report to the OHC without having to complete a checklist. The use of checklists in medicine has been fraught with many challenges including poor adherence, lack of appreciation of its importance, poor management support and oversight.^7^ Of concern was the number of staff that identified as having positive symptoms with no documentation of further action taken. This poses a great risk for staff and patient safety during a global pandemic that has seen many HCWs succumb to COVID-19.^8,9^

In principle, the concept of a self-administered screening tool is good, however, it is heavily dependent on individual understanding and motivation and line manager commitment to a safe working environment. The SARS-CoV-2 has demonstrated significant spread through fomites, especially among staff who tend to relax IPC measures when not managing patients.^10^ The practice in some wards of keeping the staff tools in a single file is problematic as it means that many people are handling the forms, increasing the risk of spread through fomites. Ideally, each staff member should keep their own form. As an alternative to handheld forms, some facilities have opted for online screening tools, but again, these are reliant on individual’s motivation to complete them. Internal quality control measures and improved oversight by line managers may alter behaviour and reduce risk if it is implemented in a team-based manner.

The KZN DoH developed a screening tool for staff that the hospital has since implemented, which requires line managers to sign off on daily screening. In addition, infrared thermometers have been purchased and are being distributed widely to all areas of the hospital so that the daily symptom screening is coupled with daily body temperature monitoring. Measuring staff members temperatures with an infrared thermometer is an objective method of screening. Although it will not detect those staff who do not contract a fever, having to manually document temperature checks may improve overall compliance. Some facilities internationally have implemented mandatory staff swabbing for SARS-CoV-2; however, although a definitive way of detecting positive staff members, it is costly and impractical in our current setting, where we have a gross backlog of tests.^11^

Of note, screening should be coupled with other aspects of IPC such as maintaining social distance, avoiding unnecessary meetings or social gatherings over lunch and tea breaks, using appropriate PPE or implementing a facility transport system for staff members so that they may avoid the use of public transport during rush hours.

## Conclusion

The approach of screening all staff, especially HCWs, for SARS-CoV-2 is a critical intervention in mitigating risk of spread. The gaps identified in this brief survey demonstrate the discrepancies between theory and practice and the flaws in using a self-screening tool to identify staff members as potential PUIs. We recommend that facilities tailor their screening programmes according to their resources, bearing in mind the potential limitations of self-screening. Such initiatives work best when local champions drive implementation, management openly endorses and monitors the intervention and staff are motivated to change their practices. Further education coupled with temperature recording using an infrared thermometer by the line manager was implemented at our facility in June 2020 and further evaluation of its efficacy will be conducted at a later date.
